# Measurement Reliability of the Remaining Dentin Thickness below Deep Carious Lesions in Primary Molars

**DOI:** 10.5005/jp-journals-10005-1478

**Published:** 2017-02-01

**Authors:** Roula Berbari, Alexandre Khairallah, Hussein F Kazan, Mohamad Ezzedine, Daniel Bandon, Elia Sfeir

**Affiliations:** 1 Clinical Chief, Department of Pediatric Dentistry, School of Dentistry Lebanese University, Beirut, Lebanon; 2 Clinical Chief, Department of Radiology, School of Dentistry, Lebanese University, Beirut, Lebanon; 3 Professor, Department of Statistics, Faculty of Science, Lebanese University, Beirut, Lebanon; 4 Professor, Department of Biology, Faculty of Science, Lebanese University, Beirut, Lebanon; 5 Professor, Department of Pediatric Dentistry, Universite De La Mediterrannee Aix Marselle II, Marseille, France; 6 Professor, Department of Pediatric Dentistry, School of Dentistry Lebanese University, Beirut, Lebanon

**Keywords:** Affected dentin, Deep carious lesions, Digital radiography, Remaining dentin.

## Abstract

**Aim:**

This study was carried out to assess the reliability of measurements of the remaining dentin thickness under deep carious lesions as estimated from digital radiographs. The goal is to allow clinicians to correlate the radiographic measurement to the exact value of the remaining dentin thickness. The results obtained will be tested further in a study that will evaluate the histopathologic pulpal state according to the caries’ lesion depth.

**Materials and methods:**

The study was conducted in the Pediatric Dentistry Department at the Lebanese University, in collaboration with the research platform of the same university. Fifty deciduous molars with deep caries on proximal surfaces liable to extraction were collected. Before extraction, a digital *in vivo* periapical radiograph was taken, followed by manual excavation of the caries. After excavation, another radiograph was taken before the tooth was sectioned through the deepest site of the lesion. Another radiograph was then obtained for each tooth fragment. To evaluate the exact thickness of the remaining dentin, each fragment was measured on a histologic macropho-tograph. The measurements were then compared statistically using a paired-samples t-test, and a correlation was sought.

**Results:**

No significant difference was observed in the radiographs between the measurement of the remaining dentin thickness before and after the excavation of caries. In contrast, the radiographic measurements of remaining dentin thickness were underestimated by an average of 20% compared with those made with macrophotographs.

**Limitations:**

Interpretation of radiographs varies from one practitioner to another and is a function of the operator’s visual acuity.

**Conclusion:**

Measuring the residual dentin thickness on a radiograph underestimates the actual thickness by about 20%. Further studies are needed to confirm these results.

**Clinical significance:**

Our results indicate that remaining dentin thickness is greater in reality than is shown on a radiograph. This information can help clinicians to refine their diagnoses and treatment plans.

**How to cite this article:** Berbari R, Khairallah A, Kazan HF, Ezzedine M, Bandon D, Sfeir E. Measurement Reliability of the Remaining Dentin Thickness below Deep Carious Lesions in Primary Molars. Int J Clin Pediatr Dent 2018;11(1):23-28.

## INTRODUCTION

Pulp vitality tests are often unreliable in pediatric dentistry.^1^ Accurate assessment of the depth of a cavity and its proximity to the pulpodentinal complex is a determining factor in the therapeutic decision, especially when pulpotomy is indicated.^2,3^

Estimation of the remaining dentin thickness (RDT) between the carious cavity and the pulp on a radiograph is crucial, and there is growing consensus that the RDT may be the most predictive measure of pulpal reactions.^4^ However, difficulties have been noted in the interpretation of radiographs, which show the location of the decay but do not specify its exact extent.^5,6^ Furthermore, radiographs are also ambiguous when it comes to precise measurement of the RDT in deep cavities.^7^

The depth of the carious cavity is frequently mentioned in the literature, mainly because the RDT is an important factor to ensure long-term clinical success in pulpal parenchyma protection. Many numerical values for the RDT are mentioned in the literature. Stanley^8^ stated that 2 mm of RDT can have a protective role in dental treatments. Thereafter, Pameijer et al^9^ reported that 1 mm or more of RDT may preserve the pulp tissue from the cytotoxic effects of zinc phosphate cements. Smith^10^ noted that, in very deep cavities with an RDT of <0.5 mm, the number and size of open dentin tubules are comparable to a real pulpal exposure. After the restoration of 49 deep cavities, Murray et al^11^ concluded that a thickness of 0.5 mm or more is needed to avoid any pulp damage. According to Bj0rndal,^12^ a carious lesion is considered deep if it involves three quarters or more of the total thickness of the dentin as seen on a radiograph. Fuks et al^13^ stated that the presence of bacteria in carious cavities with an RDT of <0.25 mm stimulates a pulpal inflammatory reaction with a significant decrease of odontoblasts and a reduction in tertiary dentin formation. All of the numerical values stated above have been estimated from radiographs.

The objective of this study was to correlate the RDT as estimated from a radiograph before extraction *(in vivo)* and after extraction and caries excavation (in *vitro).* The radiographic values thus obtained are then compared with the real histologic values as measured on a histologic macrophotograph of teeth sections. This information will allow clinicians to refine their clinical estimation of the RDT, upon which the treatment plan depends.

## MATERIALS AND METHODS

This study was conducted at the Department of Pediatric Dentistry, Dental School, Lebanese University, in collaboration with the research platform of the same university. It was carried out by two experienced instructors in the department.

The sample included 50 deciduous molars. The inclusion criteria were molars: (1) with deep proximal lesions, (2) without pulpal exposure, (3) that had not been restored, and (4) that required extraction for irreversible periodontal or endodontic lesions.

**Fig. 1:**
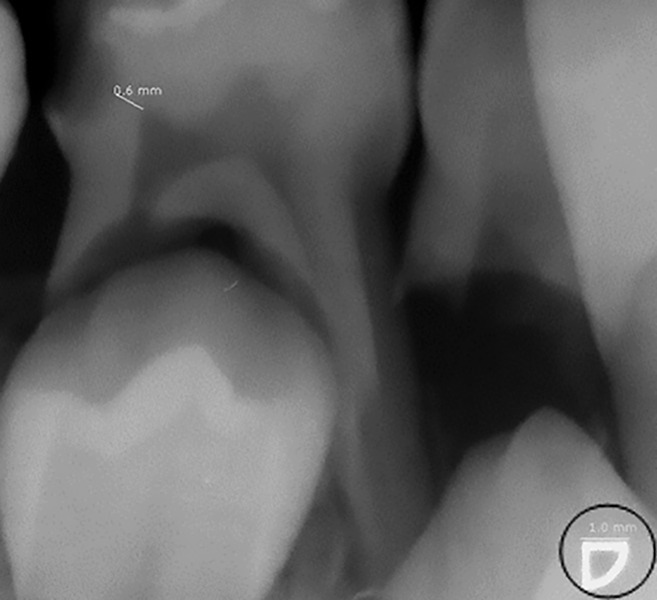
Radiograph showing the measurement of the RDT on a lower right primary first molar after software calibration (0.6 mm). The triangle circled in black in the bottom of the radiograph was used as a reference

Teeth that met the same criteria but presenting buccal or lingual carious lesions or restorations were excluded from the study to avoid any interference with radiologic readings. Before tooth extraction, a radiograph was obtained with a digital sensor (image plate Plus-Dürr Dental/number 0 plus, Germany) and a film holder (kwik-bite/Art.No.270-Kerr, Switzerland) designed for periapical radiographs. The intraoral device used was a wall-mounted X-ray scanner (Kodak 2100 Intraoral X-ray system, 60 KV-7mA, Cone 20 cm, Japan). The distance between the X-ray source and the skin (20 cm) and the exposure time (0.35 seconds) were the same for all cases. The images were processed in a digital reader preset by a professional representative of the manufacturer (VistaScan Mini Plus: Dürr Dental, Germany). For the analysis of the images, the monitor (HP L1710 17-inch LCD Monitor USA 2008) allowed visualization through the dental imaging software DBSWIN 5.4.0. After pre-calibration of the same image processing software, the RDT was measured in millimeters, taking as reference the triangle located at the corner of the image plate Durr (1 × 1 mm). A straight line was drawn between the deepest spot of the carious lesion up to the nearest spot of the pulp chamber (Fig. 1).

After extraction, the molars were stored at room temperature in a container filled with physiological serum until they were processed (<1 hour). A caries indicator (Sable seek caries indicator dark green Ultradent, USA) was then applied for 10 seconds according to the manufacturer’s indications to facilitate distinction between the different dentinal zones of the lesion.^14,15^

After rinsing with water, the infected soft dentin was entirely removed manually with an excavator (Zeffiro: ZFE004#3 Batch DO1ABA, Italy) until the hard residual dentin was reached. The affected innermost layer was kept to obtain radiographs and photographs.

The tooth was then positioned with the mesiodistal axis parallel to the sensor and in contact with it (using Sticky Wax, Orthodontic Tray Wax/Kerr), set at the level of the roots. A radiograph was taken in the same manner as before with an orthogonal incidence relative to the sensor. The distance between the X-ray tube and the teeth (22 cm) and the exposure time (0.16 seconds) were constant. The RDT was measured again using the same software.

The teeth were then sectioned in a mesiodistal direction passing through the deepest spot of the carious lesion. This step was carried out using a diamond disk 22 mm in diameter and 0.2 mm thick (Diamond Disk, Frank Dental, Germany—D.321.524.220.HP/E) mounted on a handpiece and under heavy irrigation. The tooth halves were then radiographed and RDT was measured as before.

**Fig. 2:**
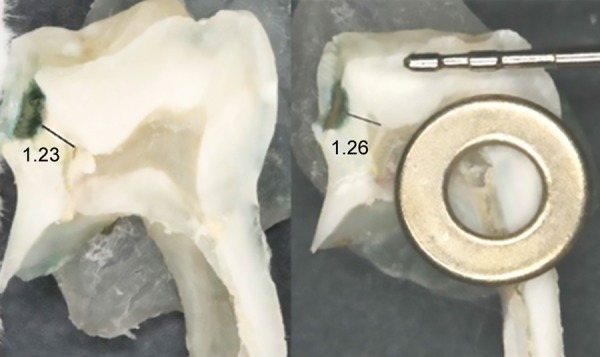
Photographs of the sectioned parts of the molar and the metal ring used as a reference with the measures of the actual thickness of the residual dentin assessed in millimeters

Finally, photographic images of the sectioned teeth were taken with a digital camera with a macro-lens in the same lighting conditions (Canon EOS 400D, MP10.1, APS-C sensor 22.2 × 14.8 mm, EF-S60, Japan) with a fixed focal distance to the tooth (14.5 cm). Before taking each photograph, a precalibrated metal ring (3 and 7 mm inner and outer diameters respectively) was placed next to the tooth as a reference when measuring the RDT (software AutoCAD 2014), as shown in Figure 2.^16^ The measuring point corresponded to that used on the corresponding radiograph and was materialized by a straight line drawn with the Windows Paint program. The average of the two measurements was recorded. All measurements were made separately by two operators. To avoid any eye strain that could distort the results and to facilitate a better reading performance, each observer made only 10 measurements over a given interval of time. Thus, we provided inter- and intraindividual reproducibility. In the case of divergence in the results, a second reading was carried out by the two operators, and the average of the measures was adopted, i.e., the traditional method of comparison by pair.

To determine whether there is a significant difference in the measurements done for the same sample at different conditions, paired Student’s t-test was done and the results were considered statistically significant when p < 0.05 (Table 1).

**Table 1:** **Table 1:** Descriptive statistics

		*A*		*B*		*C*		*D*	
Mean		2.7		2.93		2.75		3.31	
Median		2.1		2.4		2.1		2.7	
Mode		1.3		1.4		1.7		3.6	
Standard deviation		1.88		2.05		1.85		2.25	
Range		7.2		7.3		6.8		8.28	

A: Radiographic measurements before excavation; B: Radiographic measurements after excavation; C: Radiographic measurements after dental section; D: Photographic measurement after dental section

## RESULTS

**Graph 1:**
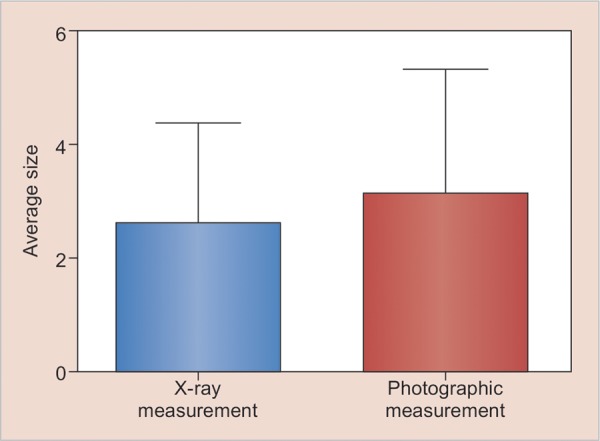
A simplified representation of the average (20%) RDT measurement between radiographs and macrophotographs of histologic sections

Our results indicated no significant difference (p = 0.676) between radiographic measurements before and after excavation (Table 2), but a highly significant difference was obtained between *in vitro* radiographs and photographic measurements (Table 3). An average underestimation of 20% in the radiographic measurements of RDT was found compared with those made by macrophotog-raphy (Graph 1).

## DISCUSSION

Dental radiography is an important tool in pediatric dentistry to measure the extent of carious lesions and their proximity to the pulp to facilitate clinical decision-making. Digital radiography has become an alternative to analog radiography because of its many advantages, including the fact that the image can be manipulated and improved with appropriate software.^17,18^

**Figs 3A and B:**
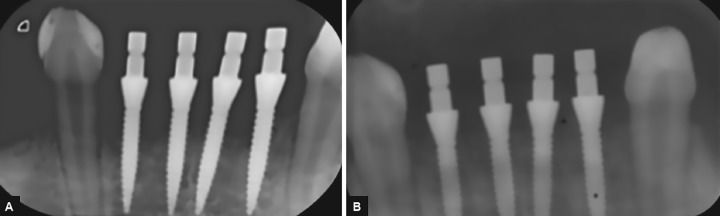
Two radiographic images taken for the same sector and at the same time. Note the change in the bone level due to modification of the contrast level

Daudibertiers et al^19^ stated that digital radiography allows better visualization of carious lesions by increasing the contrast. However, changing the contrast of the radiographs can interfere with the measurement of the dentin or bone (Fig. 3). Standardized sensors were therefore used, and precalibration of the program was carried out by a qualified representative of the manufacturer before the study commenced.^20^

**Table 2:** **Table 2:** Comparison of the RDT from radiographic measurements before and after excavation of the infected dentin

*Paired samples test*																	
		*Paired differences*															
								*95% confidence interval of the difference*									
		*Mean*		*Std. deviation*		*Std. error mean*		*Lower*		*Upper*		*t-value*		*df*		*Sig. (2-tailed)*	
Pair 1 A-B		-0.16738		2.57462		0.39727		-0.96969		0.63493		-0.421		41		0.676	

No significant difference was observed

**Table 3:** **Table 3:** Comparison of X-ray and photographic measurements after tooth section

*Paired samples test*																	
		*Paired differences*															
		*Mean*		*Std. deviation*		*Std. error mean*		*95% confidence interval of the difference*									
								*Lower*		*Upper*		*t-value*		*df*		*Sig. (2-tailed)*	
Pair 1 C-D		-0.56114		0.57447		0.08660		-0.73579		-0.38648		-6.479		43		0.000	

A highly significant difference was observed

The parallel plans technique, the use of the long cone *in vivo,* and the distance between the X-ray source and the teeth are applied in a manner that can be reproduced in all cases to obtain radiographs with great precision.^21^ We adopted the same technique of taking radiographs *in vivo* and *in vitro* for comparison because there is no significant difference between these two approaches regarding the diagnosis of proximal lesions.^22^

After sectioning the extracted tooth, we adopted the average of the two measurements obtained for each part of the tooth: the tenth of millimeter variation between the two measurements is due to the thickness of the disk and because the reference point of measurement was moved from side to side of the sectioned tooth.

Several methods of evaluation have been proposed to measure the depth of carious lesions or the RDT. Everett and Fixott^23^ and Krithika et al^24^ proposed the use of a millimeter grid superimposed on the film. To assess the depth of proximal lesions, Russell and Pitts^25^ established a scale of radiographic evaluation composed of five scores, based on the extension of the radiolucency of the dental tissues. Valizadeh et al^26^ attempted to design software to determine the depth of the decay in a more precise manner. However, the difficulties become obvious when it comes to measurement of the lesion’s depth using a radiograph and correlation of the radiographic image to the real depth of the lesion. It has been shown that in 40% of cases, dentists incorrectly estimate the depth of lesions using conventional radiographs, and in 20% of cases, they consider healthy dentin to be decayed.^27^

Similar to Jesse et al^28^ and Kooistra et al,^29^ we consider the measurement of RDT on histological sections to be the reference (gold standard). Nonetheless, our results differ from theirs and from the results of other authors, such as Lancaster et al.^7^ However, they corroborate those of Mejare and Kidd,^30^ who found an overestimation of the depth of the lesion on the radiograph, which is due to the projection of the periphery of the carious lesion on the bottom of the latter.

We explain our results as follows. The projection of the demineralized areas of the walls and the bottom of the carious cavity causes a distorted interpretation of the radiograph and an overestimation of the cavity’s depth. Because there is a superposition of more or less deminer-alized but relatively hard dentinal areas in the direction of the axis of the X-rays, we get the impression that the lesion is more profound on the radiograph.

**Figs 4A and B:**
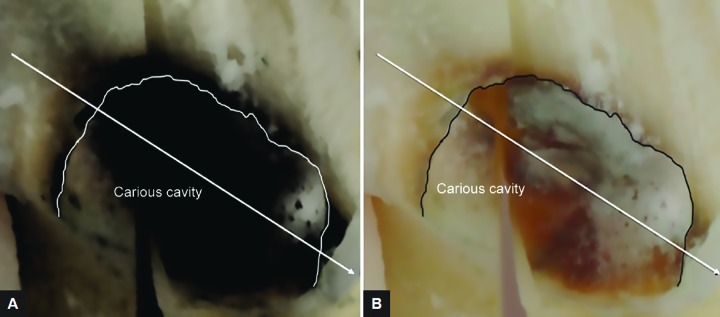
The changes in color and contrast between (A) and (B) of a horizontal cut of a carious cavity cleaned of its softened dentin show an area in black behind the tracing of the boundary of the lesion. It is this area that leads to radiographic overestimation of the depth of the carious lesion

An illustration of this situation is shown in Figure 4. These zones correspond histologically to the translucent/ demineralized zone and clinically to the leathery-to-firm dentin. When we examine a radiograph and measure the RDT, the bottom of the carious cavity is deeper than the histological reality because of the less-mineralized dentin layer that lines the bottom of the cavity.

In all cases, the diversity of the results between different studies lies in the variety of the proposed methods and the references chosen for the measurements. For example, in the study of Kamburoglu et al,^31^ the deepest spot of the carious lesion on histologic sections corresponds to the spot at which the dentin presents a different color than that of the healthy tissue. In our study, the deepest spot corresponds to the bottom of the cavity after the excavation of the caries. The underlying coloration represents the affected innermost layer of dentin characterized by the presence of altered, but not denatured, collagen.^32^

As clinicians, we usually keep the affected layer to avoid pulp exposure.^33^ Moreover, this meets the principle of “stepwise excavation” that is currently widely accepted in the restoration of deep carious lesions and is performed since many years, on primary teeth^34^; one of the main objectives of our study was to assess the RDT, thus allowing us to establish the most appropriate therapeutic indication.^7^

The nonsignificant difference between the radio-graphic measurements of the RDT before and after excavation of the infected dentin is justified by the fact that the demineralized tissues are radiolucent. Our results show an average of 20% of radiographic underestimation of the RDT compared with the measurements carried out on the macrophotographs of the histologic sections. Consequently, we argue that during the radiographic examination of a carious lesion, the operator should consider our findings before establishing a therapeutic indication.

## CONCLUSION

- It is not necessary to carry out excavation of the caries to measure the depth of the lesion.
- The measurement of the thickness of the residual dentin on the radiograph underestimates the actual thickness by approximately 20%. These results allow clinicians to refine their diagnoses. Other studies are required to further confirm these results.

### Why This Article is Important to Pediatric Dentists?

- This article permits the operator to establish a more accurate reading of radiographs in general.
- The operator is better able to estimate the real thickness of the RDT.

### What This Article adds?

When measuring the depth of the caries lesions on a radiograph, the clinician will be able to make better clinical decisions, especially in the case of deep carious primary molars that may involve pulpotomy.
